# Long-term misuse of zopiclone in an alcohol dependent woman with a history of anorexia nervosa: a case report

**DOI:** 10.1186/1752-1947-4-403

**Published:** 2010-12-10

**Authors:** Alun Morinan, Francis Keaney

**Affiliations:** 1 King's College, University of London, Institute of Psychiatry, National Addiction Centre and South London and Maudsley NHS Foundation Trust, Box 048, 4 Windsor Walk, London, SE5 8BB, UK

## Abstract

**Introduction:**

The Z-drugs, zaleplon, zopiclone and zolpidem, are short-acting hypnotics which act at the same receptor as the benzodiazepines, but seemingly without the potential for misuse and the development of dependence of the older benzodiazepines. However, with increased prescribing of Z-drugs, reports of misuse and possible dependence began to appear in the literature, particularly in people with a history of substance misuse and comorbid psychiatric illness. Here we report the case of a woman with a history of chronic zopiclone use and anorexia nervosa, admitted for alcohol detoxification.

**Case presentation:**

A 31-year old Caucasian British woman with a history of long-term zopiclone use and anorexia nervosa was admitted as an inpatient for a ten-day alcohol detoxification. Her weekly (four days out of seven) intake of alcohol was 180 units and her daily intake of zopiclone, 30 mg. Apart from a short period five years ago, she had been taking zopiclone for 13 years at daily doses of up to 90 mg. She admitted to using 'on top' of her prescribed medication, purchasing extra tablets from friends or receiving them *gratis *from her partner. After detoxification from alcohol and zopiclone, she was prescribed diazepam which she found ineffectual and voiced her intention of returning to zopiclone on leaving the hospital.

**Conclusion:**

Zopiclone is generally regarded as safer than benzodiazepines, however, this particular individual, who was using high doses of zopiclone over many years, may provide further evidence of a risk of dependency when this drug is prescribed for substance users with a comorbid psychiatric illness.

## Introduction

Insomnia is a subjective experience of poor or unrefreshing sleep that may be apparent from a delayed onset or decreased duration of sleep [1]. The short acting hypnotic cyclopyrrolone derivative, zopiclone (RP 27,267) was synthesised in the late 1970 s and introduced into clinical practice some ten years later. Zopiclone, like the other Z-drugs, zolpidem and zaleplon, interacts with the same molecular target as the benzodiazepines on the GABA_A _(γ-aminobutyric acid) receptor [2,3]. Zopiclone is available as the racemic mixture although the S-enantiomer, eszopiclone, has a 50 times higher affinity for the receptor than the R-enantiomer [4]. In terms of clinical efficacy and adverse reactions, the Z-drugs showed little difference from the short acting hypnotic benzodiazepines [5] although the fatality toxicity index (deaths/10^6 ^prescriptions) for zopiclone, 2.1, was much lower than the 9.9 for temazepam [6].

When the Z-drugs were first introduced, there was little epidemiological evidence of misuse; however, as time went on, more cases began to appear in the literature. In a report published in 1995 [7], zopiclone misuse was identified in three male teenagers attending their local CDAT (Community Drug and Alcohol Team). The zopiclone had either been prescribed or bought on the street (zim-zims) at a cost of £1 a tablet. In two individuals, zopiclone was being used to intensify and prolong the effects of alcohol while the third, a polysubstance user, was crushing up the tablets for intravenous injection. However, in none of the three was there any evidence of dependence.

In a survey of 100 clients attending a methadone maintenance program at a Liverpool clinic, six admitted to using zopiclone in daily doses up to 50 times the therapeutic dose over periods of between six and 24 months [8]. All six had previously used temazepam but preferred zopiclone because of its lack of amnesic side effects. Zopiclone was readily obtainable from people who were prescribed the drug or by forging prescriptions.

A more recent study of individuals on a methadone maintenance program in Dublin found that 37 out of 158 (23%) tested positive for a zopiclone metabolite, 2-amino-5-chloropyridine, in the urine [9]. Benzodiazepines were also present in the urine of just under 70% of this group, none of whom were currently being prescribed these drugs.

In a systematic review of the literature from 1996 to 2002, 22 cases of zopiclone abuse and 36 for zolpidem were identified with doses used up to 51 (zopiclone) and 120 (zolpidem) times higher than those recommended for insomnia [10]. The authors concluded that the two Z-drugs were relatively safe compared to the hypnotic BZs and both dependence following chronic use and addictive non-medical use were quite rare although they did warn of the possible risk to patients with a history of substance use or mental illness. This warning has been confirmed in later case reports of opioid-dependents injecting zolpidem [11] and a cocaine user snorting zaleplon obtained from several crushed capsules [12].

## Case presentation

Our patient was a 31-year-old unemployed Caucasian British woman with a diagnosis of alcohol dependence (ICD-10, F10.2) who was referred to the Acute Assessment Unit (AAU) of the hospital for a ten-day detoxification by her local CDAT. On the 28 days immediately prior to admission she had been drinking 6 L of cider (7.5% ABV) equivalent to 45 units four times a week and had been having blackouts as a result. High levels of both aspartate transaminase (AST = 86 U/L) and γ-glutamyltransferase (γGT = 187 U/L) suggested possible hepatic dysfunction but there was no evidence of cognitive impairment (MMSE score = 29). On admission, she was taking chlorpromazine (50 mg twice a day.) for anxiety, fluoxetine (40 mg once daily) for low mood and zopiclone (7.5 mg four times a day). Her physical health screen did not reveal any abnormalities.

She had a long history of both anorexia nervosa and alcohol dependence. Anorexia was first diagnosed in 1994, and when she was 17 years old she was treated as an inpatient. By the age of 18 years, her problem with alcohol had become evident and over the intervening years she has had six separate detoxifications with varying lengths of abstinence; relapses being due to life events or trauma. She also has a history of self-harm, overdosing, burning and lacerating; her last admission to Accident and Emergency was two years ago. Her father died of alcohol-related problems and her uncles are also alcohol dependent. Her sister had anorexia nervosa and died from cardiac complications, a common consequence of the severe calorific deprivation associated with this eating disorder [13].

Zopiclone (7.5 mg *nocte*) was first prescribed to help her to sleep when she was being treated for anorexia in the rehabilitation unit. She found the calming effect of taking it during the day highly desirable and when she was discharged, asked her doctor to increase the dose, claiming she had become tolerant of its hypnotic effect. She reported a typical daily intake of 60 mg, but sometimes she used up to 90 mg, beginning when she woke up and continuing throughout her waking cycle. Her use of alcohol did not change throughout the time she was taking zopiclone. Apart from prescribed zopiclone, she obtained the drug from friends (paid for) and her partner (donated).

Zopiclone was described as "her best friend" and like alcohol it gave her confidence, relaxed her and enhanced her self esteem. She said that she was ultra-possessive about her supply and would not be separated from it, keeping it with her at all times. During the 13 years of using there had only been one relatively short period of abstinence, which occurred six years ago, when she was in the hospital for an alcohol and zopiclone detoxification. However, this ended with the recurrence of the anorexia and she was prescribed zopiclone to help her sleep.

The current detoxification followed the standard protocol used in the AAU, namely a tapering off of doses of chlordiazepoxide (130 mg to zero over six days) and on each of the first five days, an i.m. injection of Pabrinex^® ^and thereafter Vitamin B Compound Strong tablets. Her zopiclone was reduced from 7.5 mg *nocte *via 3.75 mg to zero over the same period as the chlordiazepoxide after which she was started on diazepam 20 mg, with the dose being reduced incrementally by 1 mg each day. She found diazepam an ineffectual substitute and had cravings for zopiclone and said she could not wait to return to taking it as soon as possible. She had no intention of stopping zopiclone in the forseeable future.

Our patient is still off zopiclone (and alcohol) after 17 months although she continues to have strong cravings for zopiclone (more so than for alcohol) which would be easy to satisfy. She is concerned that zopiclone is not considered addictive and that there is no specific protocol for detoxification, other than substitution with diazepam, and help for understanding this addiction and preventing relapse.

## Discussion

After two decades of clinical use, the Z-drugs are considered to have a relatively low risk of being misused or producing dependence compared to benzodiazepine hypnotics like temazepam. This was the conclusion reached by Hayak *et al*. [10] on the basis of the number of global prescriptions and a systematic *Medline *search covering a seven-year period up to 2002. However, the authors cautioned against their uncritical use in patients with a history of substance misuse and/or psychiatric illness. A similar note of caution was sounded by Cimolai [14] who identified 20 cases of dependence on zopiclone (dose range 7.5 to 390 mg/d) in Canada between 1991 and 2006.

Dependence on alcohol may co-exist with dependence on prescribed psychotropic drugs, thus complicating clinical treatment. Johansson *et al*. [15] collected data from a sample of alcohol dependents in both community (n = 130) and inpatient (n = 23) settings in Sweden. Seven percent of the outpatients and 13% of the inpatients were found to be dependent on zopiclone or zolpidem compared to 12% and 30% respectively for benzodiazepines. However, the severity of dependence as measured relative to the defined daily dose (DDD for zopiclone = 7.5 mg, zolpidem = 10 mg) was between 1.0 and 1.5 (range of mean values for the two Z-drugs in the two treatment settings) whereas for the benzodiazepines, the values were 5.5 for outpatients and 11.5 for inpatients; a value of ≥4.0 being considered high dose dependence (HDD).

Using these values, our patient would be defined as HDD with a value of 12. In other case studies on zopiclone dependence, ranges of 2 to 40 [9] and 1 to 51 [10] have been noted. In one case described as ultrahigh dose dependence, a 34-year old woman was taking zolpidem equivalent to 200 and when abruptly discontinuing the drug, suffered withdrawal seizures [16].

In the Dublin sample, the mean age of onset and duration of zopiclone use was 28 and 4.2 years respectively with the mean age of the cohort being 32 years [9]. From the review of the 22 cases of zopiclone dependence, where the individual ages were presented (n = 15), the mean age was 39 years [10]. Our patient was 31 years old and although there are a few case reports of zopiclone being taken at doses higher than her typical daily intake of 60 mg, the 13 years of almost continuous use might be considered particularly unusual. It is also interesting to note that she had every intention of returning to zopiclone as soon as possible after discharge and that her experience with benzodiazepines was considered inferior to zopiclone. Comorbidity of alcohol dependence and anorexia nervosa is common and a serotonergic neuronal dysfunction has been implicated in both disorders [17]. In our patient, her cravings for zopiclone following withdrawal and interim substitution with diazepam would be indicative of dependence. We would recommend a cautious approach to prescribing Z-drugs to patients with a dual diagnosis and that particular attention be paid to any request for an increase in the daily dose.

## Conclusion

We have described an individual with an atypically long history of high dose dependence on zopiclone, alcohol dependence and anorexia nervosa. This case adds further support to the growing body of evidence that prescribing zopiclone to drug users with an underlying psychiatric disorder should be carefully monitored.

## Patient's perspective

Gratefully I'm still off zopiclone though I am aware I could get hold of it easily. As yet, there's no detox for coming off zopiclone. No help provided for the mental or physical reasons and withdrawal.

## Consent

Written informed consent was obtained from the patient for publication of this case report. A copy of the written consent is available for review by the Editor-in-Chief of this journal.

## Competing interests

The authors declare that they have no competing interests.

## Authors' contributions

FK is the Consultant Psychiatrist on the AAU and AM is a Research Scientist (Pharmacology). AM interviewed the patient and wrote the manuscript. Both authors read and approved the final manuscript.
